# Hepatic arterial infusion chemotherapy combined with lenvatinib and toripalimab for large hepatocellular carcinoma (> 10 cm) with major portal vein tumor thrombosis: a multicenter propensity score matching analysis

**DOI:** 10.3389/fimmu.2025.1638173

**Published:** 2025-10-24

**Authors:** Yangyang Li, Danchen Wang, Fengtao Zhang, Xiang Zheng, Yipei Song, Yang Ran, Xiangran Cai

**Affiliations:** ^1^ Department of Radiology, The First Affiliated Hospital of Jinan University, Guangzhou, Guangdong, China; ^2^ Vascular Interventional Surgery, Shenzhen Nanshan People’s Hospital, Shenzhen, Guangdong, China; ^3^ Department of Interventional Therapy, Zhuhai People’s Hospital(Zhuhai Clinical Medical College of Jinan University), Zhuhai, Guangdong, China; ^4^ Department of Radiology, The Second Affiliated Hospital of Nanchang University, Nanchang, Jiangxi, China; ^5^ Guangdong Provincial Key Laboratory of Optical Fiber Sensing and Communications, Institute of Photonics Technology, Jinan University, Guangzhou, Guangdong, China; ^6^ College of Physics & Optoelectronic Engineering, Jinan University, Guangzhou, China

**Keywords:** hepatocellular carcinoma, hepatic artery infusion chemotherapy, lenvatinib, toripalimab, high tumor burden, portal vein tumor thrombosis, propensity score matching

## Abstract

**Background:**

Portal vein main trunk tumor thrombus is one of the most intractable complications of hepatocellular carcinoma(HCC), often occurring in patients with high intrahepatic tumor burden(>10 cm). High tumor burden HCC complicated by portal vein main trunk tumor thrombus is regarded as the very advanced stage with extremely poor therapeutic efficacy and very limited treatment options and its long-term survival depends on the dual remission of intra-hepatic tumors and tumor thrombi. Previous phase III trials have confirmed the ability of HAIC to effectively relieve high tumor burden HCC, yet HAIC alone cannot effectively manage tumor thrombi and intrahepatic progression. The efficacy of the combination of HAIC, lenvatinib and toripalimab in advanced HCC has also been confirmed by existing clinical evidence. Therefore, the combination of HAIC, lenvatinib and toripalimab may be a potentially effective treatment regimen for high tumor burden HCC complicated by portal vein main trunk tumor thrombus.

**Methods:**

A retrospective review was conducted on the clinical data of patients with high tumor burden HCC complicated by main portal vein tumor thrombus who received HAIC combined with lenvatinib and toripalimab(HAICLT group) or HAIC alone(HAIC group) from August 2019 to December 2023. Propensity score matching was employed to balance the baseline differences between the groups. The overall survival time, progression-free survival time, objective response rate, and disease control rate were compared between the groups.

**Results:**

After PSM, the median OS and median PFS of the HAICLT group were 21.2 months and 7.4 months respectively, significantly better than 6.6 months(HR: 0.35; 95% CI: 0.22-0.56, P < 0.001) and 3.0 months(HR: 0.45; 95% CI: 0.31-0.66, P < 0.001) of the HAIC group. In terms of treatment response, the HAICLT group also accomplished higher rates of intrahepatic responses(ORR: 57.7% vs 19.7%, P<0.001; DCR: 91.5% vs 59.2%, P<0.001) and PVTT responses(ORR: 62.0% vs 21.1%, P<0.001; DCR: 93.0% vs 50.7%, P<0.001) compared to the HAIC group. No significant statistical differences were found in the incidence rates of adverse events at all grades and grades 3–4 between the groups.

**Conclusion:**

Compared with HAIC alone, the combination of HAIC, lenvatinib, and toripalimab can effectively prolong the survival prognosis of patients with large HCC complicated by major PVTT and achieve intrahepatic and PVTT remission. It is a promising treatment approach.

## 1 Introduction

Hepatocellular carcinoma(HCC) is the fourth most common solid tumor and the third leading cause of cancer-related death worldwide with 9.5% of global incidence and only 14.1% five-year survival rate, characterized by high tumor heterogeneity and invasiveness (1–3). Portal vein tumor thrombus(PVTT) formation is a major and thorny complication of HCC, with an incidence rate ranging from approximately 44.0% to 62.2% (4). Particularly in cases of major PVTT [first branch portal vein invasion(Vp3) or main trunk portal vein invasion(Vp4)], the prognosis is extremely poor and the available treatment options are limited (5). Research has indicated that the occurrence of portal vein involvement is positively correlated with tumor diameter. Compared with small HCCs(<3 cm), the incidence of PVTT in large HCCs(5–10 cm) increases by 25.2% (6). Currently, it has become common for HCC to be concurrently associated with high tumor burden(>10 cm) and major PVTT. Unfortunately, sorafenib, as the standard treatment, has a modest efficacy, only extending the overall survival(OS) by less than 2 months compared with placebo (7). At present, the treatment of a special type of HCC with major PVTT and a tumor diameter greater than 10 cm poses a great challenge.

Tumor characteristics, including diameter, quantity, tumor thrombus, and extra - hepatic metastasis, contribute to the heterogeneity of HCC, which is also the primary factor leading to poor prognosis of HCC (6). For large HCC complicated with major PVTT, achieving long-term survival requires focusing on the remission of intrahepatic lesions and PVTT, and then seeking opportunities for radical treatment. Hepatic arterial infusion chemotherapy(HAIC) is a local chemotherapy method optimized from traditional systemic chemotherapy. By maintaining a high local concentration of chemotherapy drugs while effectively reducing side effects, it can effectively kill tumor cells. Two previous phase III trials reported satisfactory survival prognosis and tumor - shrinking ability of HAIC, whether compared with TACE or sorafenib (8, 9). According to the guidelines of the Japan Society of Hepatology(JSH), HAIC has been recommended as one of the preferred treatment options for HCC complicated with PVTT (10). However, HAIC alone cannot effectively manage advanced HCC, let alone the extremely advanced type of large HCC complicated with major PVTT.

According to the Barcelona Clinic Liver Cancer(BCLC) staging system, regardless of the size and number of tumors, the occurrence of PVTT is classified as advanced - stage, and systemic treatment is recommended as the first-line option. With the popularization of the concept of combination therapy, the combination of vascular - based local treatment and systemic treatment has been increasingly applied clinically in the treatment of HCC. The REFLECT trial reported that lenvatinib was non-inferior to sorafenib in the treatment of unresectable HCC, and thus lenvatinib was approved as one of the first-line treatments for advanced HCC (11). As the treatment of HCC has entered the era of immunotherapy, the efficacy of targeted therapy combined with immunotherapy has been widely studied and confirmed (12, 13). Interestingly, although the efficacy of a single immunosuppressant is not ideal, with an effective rate of less than 30%, it often plays a crucial role in combination therapy (14). Based on the concept of personalized treatment in precision medicine, achieving dual remission of intra-hepatic lesions and PVTT through the combination of local interventional therapy and systemic targeted immunotherapy may be a potential opportunity to obtain long-term survival. A previous randomized trial and a retrospective study reported the efficacy of HAIC combined with lenvatinib and toripalimab in high-risk HCC and advanced HCC respectively, and both obtained satisfactory positive results (15, 16). Currently, there is no effective treatment regimen for HCC with major PVTT and a diameter exceeding 10 cm, and there are no relevant reports.

As of now, the efficacy of the combined application of HAIC, lenvatinib, and toripalimab in the treatment of HCC with PVTT and a diameter exceeding 10 cm remains an uncharted territory. Therefore, this study aims to explore the safety and efficacy of HAIC combined with lenvatinib and toripalimab in the treatment of HCC with diameter over 10 cm and major PVTT.

## 2 Materials and methods

### 2.1 Study population

This retrospective, multicenter cohort study was conducted in adherence to the principles outlined in the Declaration of Helsinki and was ethically approved by the institutional review boards of the first affiliated hospital of Jinan university. Informed consent for surgical treatment was obtained from all enrolled patients prior to their procedures.

From March 2019 to November 2023, clinical data of HCC with diameter larger than 10cm and major PVTT received HAIC combined with lenvatinib plus toripalimab treatment or HAIC alone at four medical centers in China were retrospectively reviewed and collected. All enrolled patients were diagnosed with HCC according to the diagnostic criteria established by the American Association for the Study of Liver Diseases(AASLD) or the European Association for the Study of the Liver(EASL), with histopathological confirmation obtained through liver biopsy for cases with diagnostic uncertainties. The inclusion criteria were as follows: (1) age between 18 and 80 years; (2) largest tumor diameter ≥ 10cm; (3) PVTT classified as type Vp3 or Vp4; (4) Eastern Cooperative Oncology Group(ECOG) Performance Status of 0; (5) liver function categorized as Child-Pugh A-B or ALBI 1-2. The exclusion criteria included: (1) missing clinical or imaging data; (2) prior treatment for HCC before arterial therapy; (3) loss to follow-up exceeding six months; and (4) coexisting other malignancies.

### 2.2 Treatment procedure and follow-up

All vascular interventional treatments were performed by two or more experienced interventional imaging physicians under digital subtraction angiography(DSA) guidance achieving technical success.

HAIC procedure: The procedure for Hepatic artery catheterization are as follows: Following femoral artery puncture via the modified Seldinger technique, a 5F vascular sheath was inserted. Through this sheath, a 5F Yashiro catheter(Terumo, Tokyo, Japan) was introduced for angiography of the superior mesenteric artery and celiac trunk in sequence. This was aimed at precisely discerning the origins of the intra- and extra-hepatic arteries that supply the tumor. Subsequently, a 2.7Fr microcatheter system(Terumo Corporation, Tokyo, Japan) was placed.

mFOLFOX6 - HAIC protocol: Oxaliplatin(85 mg/m^2^) was given via 2-hour infusion; leucovorin(400 mg/m^2^) was administered via 2-hour infusion; 5-FU(400 mg/m^2^) was given as a bolus, followed by continuous infusion of either 2400 mg/m^2^ over 46 hours or 1200 mg/m^2^ over 23 hours. HAIC was repeated every 3–4 weeks. Dose adjustments were implemented when persistent or severe treatment-related adverse reactions occurred, with therapy resumed once the patient condition stabilized. The HAIC regimen was repeated every three weeks. Post-treatment, a full abdominal enhanced CT scan was conducted every eight weeks to assess treatment efficacy.

Lenvatinib (Lenvima, Tokyo, Japan) and toripalimab (Tuoyi, Shanghai, China) was administered within 3 days following the initial HAIC treatment. The dosage was set at 12 mg/day for patients weighing > 60 kg, and 8 mg/day for those weighing< 60 kg. In the event of severe and intolerable adverse reactions, the HAIC dose reduction and toripalimab discontinue were allowed. Once the adverse reactions resolved or disappeared, the initial dose was gradually resumed. Lenvatinib was discontinued 4 days before the next HAIC cycle and resumed 3 days afterward. If the suspension lasted longer than one month, the patient was excluded from the study. The treatment cycle was 4 weeks.

All recruited patients undergo follow-up evaluations one month after the initial treatment and subsequently every three months. The follow-up assessments include a comprehensive physical examination, evaluation of liver function, measurement of serum alpha-fetoprotein(AFP) levels, additional biochemical blood tests, and enhanced abdominal CT or MRI scans. Chest CT scans and positron emission tomography computed tomography(PET-CT) scans, as well as any other imaging studies deemed clinically necessary, are selectively conducted based on clinical decision-making by the treating physicians.

### 2.3 Study endpoints

The primary endpoint of this study is OS, defined as the time interval from the initial definitive diagnosis of HCC to the latest follow-up or clinical death. The secondary endpoint is PFS, calculated as the time interval from the initial definitive diagnosis of HCC to the first assessment of disease progression(PD) according to the modified Response Evaluation Criteria in Solid Tumors 1.1(mRECIST 1.1) criteria. Tumor response is evaluated by two experienced radiologists according to the mRECIST 1.1 criteria and is classified as complete response(CR), partial response(PR), stable disease(SD), or PD. The third endpoint includes the intrahepatic and PVTT objective response rate(ORR), which is the proportion of cases with tumor responses classified as CR and PR, and the disease control rate(DCR) is the proportion of cases with tumor responses classified as CR, PR, and SD. Treatment-related adverse events are recorded according to the Common Terminology Criteria for Adverse Events 5.0(CTCAE 5.0) standards.

### 2.4 Propensity score matching

1:1 propensity score matching(PSM) were utilized to mitigate baseline disparities. The tolerance level for propensity matching was established at 0.02. The covariates incorporated into the balancing process encompassed age, gender, HBV infection status, cirrhosis presence, ascites, Child-Pugh classification, ALBI grade, AFP levels, maximum tumor diameter, tumor burden and metastasis.

### 2.5 Statistical analysis

Statistical analyses were conducted using R software(Rstudio version 4.4.1) and SPSS(IBM SPSS Statistics 26, USA). For continuous variables that followed a normal distribution, results are presented as mean ± standard deviation and analyzed using Student’s t-test. For non-normally distributed variables, medians were utilized and evaluated using the Mann-Whitney U test. Categorical variables are reported as percentages and assessed using either the chi-square test or Fisher’s exact test. Survival analysis was performed using the Kaplan-Meier method, and Kaplan-Meier curves were generated for OS and PFS across the overall cohort and the PSM cohort. A Cox proportional hazards regression model was employed to identify independent prognostic factors influencing OS and PFS.

A two-tailed P-value of less than 0.05 was considered statistically significant.

## 3 Results

### 3.1 Patient characteristics and treatment

**Figure 1:**
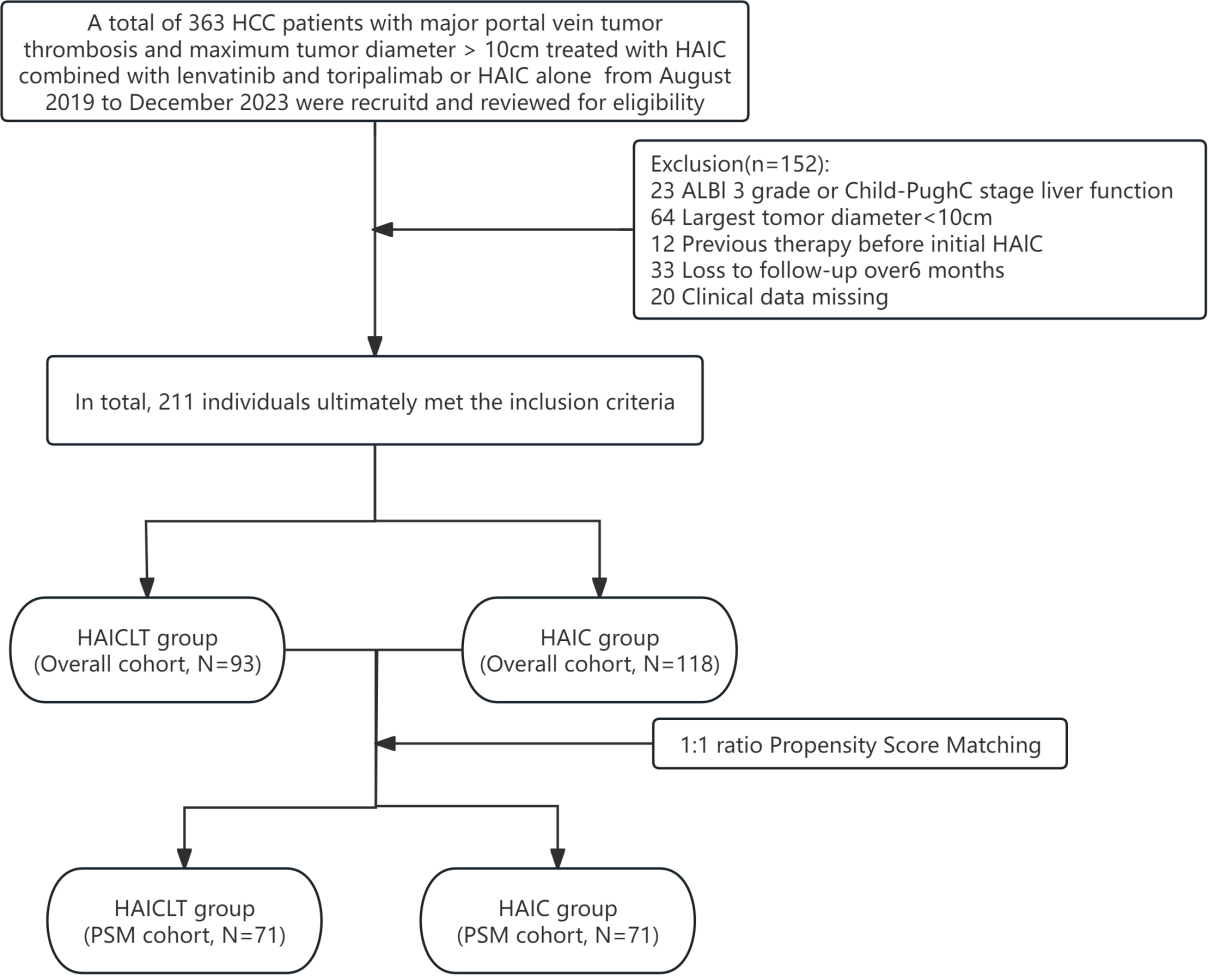
Flowchart of the patients selection process.

After a thorough eligibility screening, 93 large HCC with major PVTT patients who received HAIC combined with lenvatinib plus toripalimab treatment, along with 118 who only received HAIC treatment were ultimately included. The flowchart of the eligibility screening process is presented in **Figure 1**.

In the overall cohort, the majority of the enrolled patients were males with hepatitis B virus-related HCC. The mean largest tumor diameters in the combination - therapy group and the monotherapy group were 12.8 ± 3.7cm and 12.3 ± 4.1cm respectively. Compared with the HAIC group, the HAICLT group enrolled a significantly higher proportion of patients with ECOG PS 0 and ALBI grade 1 liver function. After 1:1 PSM to balance the baseline differences, the aforementioned differences between the groups disappeared, forming a PSM cohort containing 73 pairs. The detailed baseline characteristics of all the selected patients before and after PSM are presented in **Table 1**.

**Table 1:** The patients baseline characteristic before and after propensity score matching.

Covariate	Overall cohort			PSM cohort		
	HAICLT group (n=93)	HAIC group (n=118)	P value	HAICLT group (n=71)	HAIC group (n=71)	P value
Gender			0.123			1.000
Male	83(89.2%)	112(94.9%)		65(91.5%)	65(91.5%)	
Female	10(10.8%)	6(5.1%)		6(8.5%)	6(8.5%)	
Age^a^	48.8 ± 11.0	51.6 ± 11.5	0.260	±	±	0.754
≤65y	85(91.4%)	102(86.4%)		65(91.5%)	66(93.0%)	
>65y	8(8.6%)	16(13.6%)		6(8.5%)	5(7.0%)	
ECOG PS			0.017			1.000
0	84(90.3%)	92(78.0%)		63(88.7%)	63(88.7%)	
1	9(9.7%)	26(22.0%)		8(11.3%)	8(11.3%)	
Comorbidities			0.491			1.000
Presence	14(15.1%)	22(23.7%)		10(14.1%)	10(14.1%)	
Absence	79(84.9%)	96(76.3%)		61(85.9%)	61(85.9%)	
HBsAg			0.390			0.546
Presence	86(92.5%)	105(89.0%)		66(93.0%)	64(90.1%)	
Absence	7(7.5%)	13(11.0%)		5(7.0%)	7(9.9%)	
Cirrhosis			0.057			0.494
Presence	69(74.2%)	100(84.7%)		58(81.7%)	61(85.9%)	
Absence	24(25.8%)	18(15.3%)		13(18.3%)	10(14.1%)	
Ascites			0.309			0.566
Presence	27(29.0%)	27(22.9%)		17(23.9%)	20(28.2%)	
Absence	66(71.0%)	91(77.1%)		54(76.1%)	51(71.8%)	
Child-Pugh grade			0.710			0.494
A	79(84.9%)	98(83.1%)		58(81.7%)	61(85.9%)	
B	14(15.1%)	20(16.9%)		13(18.3%)	10(14.1%)	
ALBI grade			0.008			0.864
1	45(48.4%)	36(30.5%)		29(40.8%)	28(39.4%)	
2	48(51.6%)	82(69.5%)		42(59.2%)	43(60.6%)	
AFP			0.921			0.700
≤400ng/L	27(29.0%)	35(29.7%)		19(26.8%)	17(23.9%)	
>400ng/L	66(71.0%)	83(70.3%)		52(73.2%)	54(76.1%)	
ALB^b^(g/L)	37.8(35.1-43.3)	39.0(36.1-43.3)	0.243	38.7(35.1-44.3)	39.0(36.1-45.1)	0.561
ALT^b^(U/L)	44.2(32.5-64.9)	45.3(31.2-69.0)	0.386	43.8(31.2-65.8)	44.4(31.5-68.1)	0.638
AST^b^(U/L)	76.2(49.2-127.2)	77.2(51.4-122.5)	0.852	77.6(49.2-137.9)	77.2(51.6-122.7)	0.889
TBIL^b^(umol/l)	18.1(13.2-24.6)	17.3(12.2-25.3)	0.161	18.1(14.2-23.7)	17.7(11.8-24.6)	0.432
Large tumor diameter^a^ (cm)	12.8 ± 3.7	12.3 ± 4.1	0.438	12.5 ± 3.9	12.2 ± 4.0	0.537
Tumor number			0.619			0.389
1-3	41(44.1%)	48(40.7%)		30(42.2%)	25(35.2%)	
>3	52(55.9%)	70(59.3%)		41(57.8%)	46(64.8%)	
PVTT			0.509			
Vp3	36(38.7%)	51(43.2%)		28(39.4%)	29 (40.8%)	0.864
Vp4	57(61.3%)	67(56.8%)		43(60.6%)	42(59.2%)	
Extrahepatic metastasis			0.474			0.614
Presence	44(47.3%)	50(42.4%)		35(49.3%)	38(53.5%)	
Absence	49(52.7%)	68(57.6%)		36(50.7%)	33(46.5%)	

P-value < 0.05 indicated a significant difference.

^a^Data are means ± standard deviations.

^b^Data are medians, with interquartile ranges in parentheses.

HAICLT, Hepatic arterial infusion chemotherapy combined with lenvati**n**ib plus toripalimab therapy; HAIC, Hepatic arterial infusion chemotherap; PSM, Propensity Score Matching; ECOG PS, Eastern Cooperative Oncology Group Performance Status; HBsAg, Hepatitis B surface antigen; ALBI, Albumin-bilirubin; AFP, α-fetoprotein; ALB,: Albumin; ALT, alanine aminotransferase; AST, aspartate aminotransferase; TBIL, total bilirubin; PVTT: Portal vein tumor thrombosis.

### 3.2 Comparison of survival outcomes

The median follow-up time was 18.9 months. In the overall cohort, the median OS of the HAICLT group and the HAIC group were 21.2 months and 7.5 months respectively(Hazard Ratio(HR): 0.35; 95% Confidence Interval(CI): 0.24-0.53, P < 0.001) and the median PFS were 6.7 months and 3.7 months respectively(HR: 0.55; 95% CI: 0.40-0.76, P < 0.001). The 6-month 12-month and 18-month OS rates of the HAICLT group and the HAIC group were 91.7%, 69.2%, 52.9% and 75.7%, 17.9%, 6.0% respectively (P < 0.001) and the 3-month 6-month and 12-month PFS rates were 82.5%, 59.3%, 12.5% and 56.5%, 23.8%, 0.4% respectively (P < 0.001).

**Figure 2:**
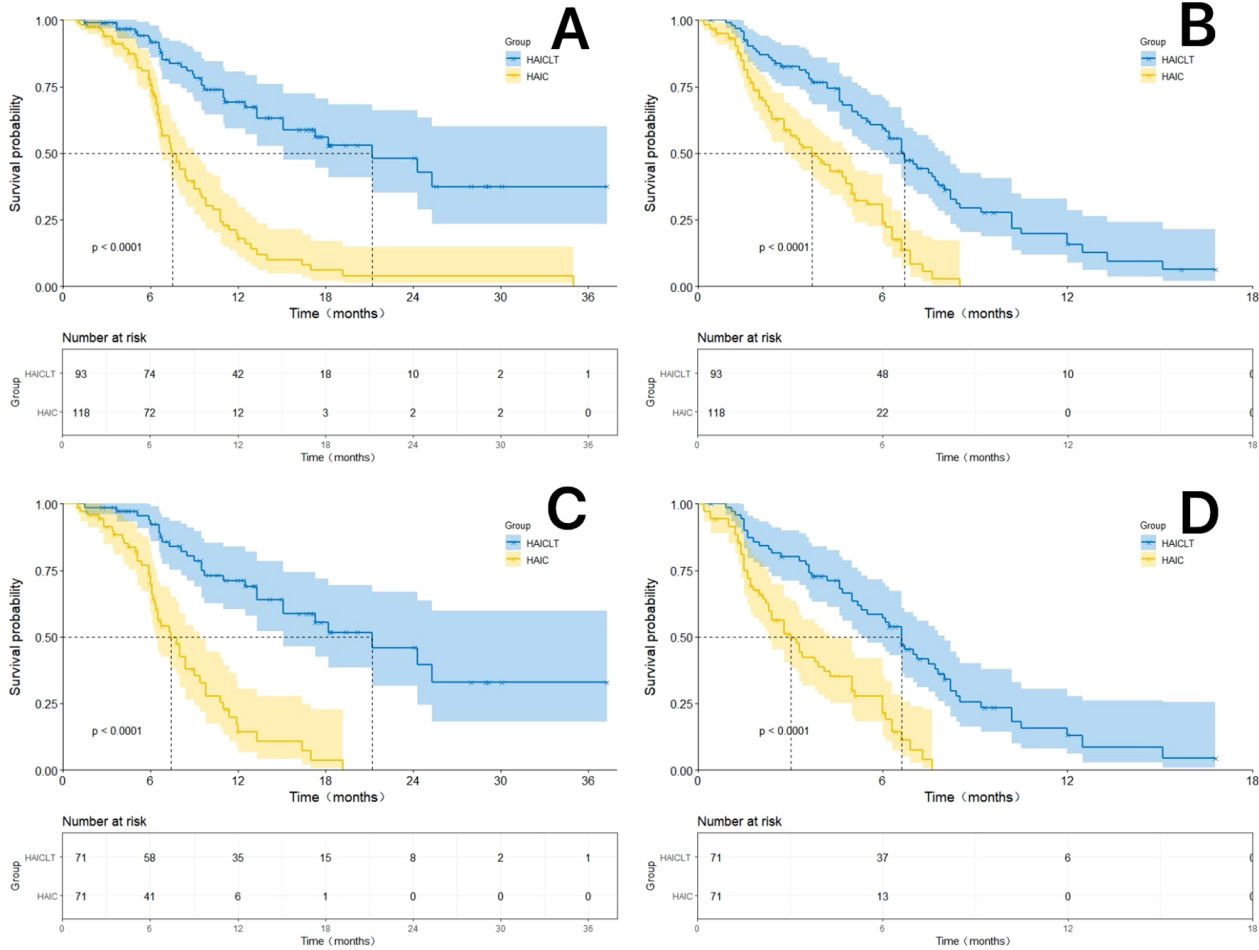
The Kaplan-Meier survival curves by Log-rank test for the HAICLT group and the HAIC group with or without propensity score matching(PSM), inverse probability of treatment weighting adjustment and coarsened exact matching. **(A)** The Kaplan-Meier curves comparing the overall survival between the HAICLT group and the HAIC group without PSM-adjusted; **(B)** The Kaplan-Meier curves comparing the overall survival between the HAICLT group and the HAIC group without PSM-adjusted; **(C)**. Comparison of PSM-adjusted overall survival between the HAICLT group and HAIC groups; **(D)** Comparison of PSM-adjusted progression-free survival between the HAICLT group and HAIC groups.

In the PSM cohort, the HAICLT group still demonstrated superior survival benefits. The median OS and median PFS of the HAICLT group were 21.2 months and 7.4 months respectively which were also significantly better than 6.6 months(HR: 0.35; 95% CI: 0.22-0.56, P < 0.001) and 3.0 months(HR: 0.45; 95% CI: 0.31-0.66, P < 0.001) of the HAIC group. The 6-month 12-month and 18-month OS rates and the 3–month, 6-month and 12-month PFS rates of the HAICLT group were 92.3%, 71.1%, 52.7% and 78.5%, 56.9%, 13.0% respectively. The 6-month 12-month and 18-month OS rates and the 3-month 6-month and 12-month PFS rates of the HAIC group were 69.4%, 14.2%, 3.5% and 49.3%, 21.3%, 0.2% respectively(all P<0.001). The Kaplan-Meier survival curves before and after PSM are shown in **Figure 2**.

### 3.3 Tumor response

The responses of intrahepatic lesions and PVTT before and after PSM are presented in **Table 2**. According to the mRECIST 1.1, in the overall cohort, the HAICLT group achieved significantly higher proportions of intrahepatic responses(ORR: 58.1% vs 20.3%, P < 0.001; DCR: 92.5% vs 61.9%, P<0.001) and PVTT responses(ORR: 67.7% vs 23.7%, P<0.001; DCR: 94.6% vs 55.1%, P<0.001) than the HAIC group. In the combination therapy group, 6 cases achieved CR and 48 cases achieved PR, with 14 cases(15.1%) underwent conversion surgery. In contrast, in the HAIC group, the majority of patients experienced PD of intrahepatic lesions(38.1%) and PVTT(44.9%). In terms of overall response, the HAICLT group was significantly superior to the HAIC group both before and after PSM(P < 0.001). Similarly, after PSM, the HAICLT group also accomplished higher rates of intrahepatic responses(ORR: 57.7% vs 19.7%, P<0.001; DCR: 91.5% vs 59.2%, P<0.001) and PVTT responses(ORR: 62.0% vs 21.1%, P<0.001; DCR: 93.0% vs 50.7%, P<0.001) compared to the HAIC group, and 15.5% of the patients successfully underwent surgical conversion.

**Table 2:** The best intrahepatic and PVTT response before and after propensity score matching.

Best Response	Overall cohort			PSM cohort		
	HAICLT group(n=93)	HAIC group (n=118)	P value	HAICLT group(n=71)	HAIC group (n=71)	P value
Intrahepatic tumor			< 0.001			< 0.001
CR	6(6.5%)	0(0%)		5(7.0%)	0(0%)	
PR	48(51.6%)	24(20.3%)		36(50.7%)	14(19.7%)	
SD	32(34.4%)	49(41.5%)		24(33.8%)	28(39.4%)	
PD	7(7.5%)	45(38.1%)		6(8.5%)	29(40.8%)	
ORR	58.1%(54/93)	20.3%(24/118)	< 0.001	57.7%(41/71)	19.7%(14/71)	< 0.001
DCR	92.5%(86/93)	61.9%(73/118)	< 0.001	91.5%(65/71)	59.2%(42/71)	< 0.001
PVTT						
CR	12(12.9%)	0(0%)	< 0.001	8(11.3%)	0(0%)	< 0.001
PR	51(54.8%)	28(23.7%)		36(50.7%)	15(21.1%)	
SD	25(26.9%)	37(31.4%)		22(31.0%)	21(29.6%)	
PD	5(5.4%)	53(44.9%)		5(7.0%)	35(49.3%)	
ORR	67.7%(63/93)	23.7%(28/118)	< 0.001	62.0%(44/71)	21.1%(15/71)	< 0.001
DCR	94.6%(88/93)	55.1%(65/118)	< 0.001	93.0%(66/71)	50.7%(36/71)	< 0.001
Conversion to resection	15.1%(14/93)	5.1%(6/118)	< 0.001	15.5%(11/71)	4.2%(3/71)	< 0.001

PSM, Propensity Score Matching; HAICLT, Hepatic arterial, infusion chemotherapy combined with lenvatinib plus toripalimab therapy; HAIC, Hepatic arterial infusion chemotherap; CR, Complete response, PR, partial response, SD, Stable disease, PD, Progressive disease, ORR, Objective response rate, DCR, Disease control rate, PVTT, protal vein tumor thombsis.

### 3.4 Prognosis related risk factors and subgroup analysis

Univariate analysis revealed that PVTT type and treatment regimen were significant independent factors associated with OS(P < 0.1). Independent risk factors associated with PFS included PVTT type, extrahepatic metastasis and treatment regimen. Multivariate analysis incorporating all these factors demonstrated that PVTT type Vp3 and receiving HAIC combined with lenvatinib plus toripalimab was an independent factor for longer OS. Meanwhile, absence of PVTT type Vp3, extrahepatic metastasis and receiving HAIC combined with lenvatinib plus toripalimab were associated with better PFS. The risk factors associated with OS and PFS in univariate and multivariate analyses are shown in **Table 3**.

**Table 3:** Predictors for overall survival and progression-free survival based on univariate and multivariate analysis.

Factors	Overall survival				Progression-free survival			
	Univariate analysis	Multivariate analysis			Univariate analysis	Multivariate analysis		
	P value	HR	95%CI	P value	P value	HR	95%CI	P value
Gender	0.584	–	–	–	0.199	–	–	0.
Male								
Female								
Age	0.414	–	–	–	0.643	–	–	–
≤65y								
>65y								
ECOG PS	0.100	–	–	–	0.411	–	–	–
0								
1								
Comorbidities	0.704	–	–	–	0.253	–	–	0.
Presence								
Absence								
HBsAg	0.386	–	–	–	0.963	–	–	–
Presence								
Absence								
Crrihosis	0.766	–	–	–	0.151	–	–	–
Presence								
Absence								
Ascites	0.102	–	–	–	0.246	–	–	–
Presence								
Absence								
Child-Pugh grade	0.128	–	–	–	0.305	–	–	–
A								
B								
ALBI grade	0.113	–	–	–	0.254	–	–	–
1								
2								
AFP	0.113	–	–	–	0.475	–	–	–
≤400ng/mL								
>400ng/mL								
Tumor number	0.795	–	–	–	0.113	–	–	–
1-3								
>3								
PVTT	**0.012**	**0.32**	**0.18**	**0.025**	**0.025**	**0.40**	**0.15-0.75**	**0.032**
Vp3								
Vp4								
Extrahepatic metastasis	0.753	–	–	–	**0.005**	**0.41**	**0.24 – 0.66**	**0.015**
Presence								
Absence								
Treatment regimen	**<0.001**	**0.24**	**0.16 - 0.37**	**< 0.001**	**< 0.001**	**0.35**	**0.24 -0.50**	**< 0.001**
HAICLT								
HAIC								

HR, Hazard ratios; CI, Confidence interval; ECOG PS, Eastern cooperative oncology group performance status; HBsAg, Hepatitis B surface antigen; ALBI, Albumin-bilirubin ratio; AFP:α-fetoprotein; PVTT, Portal vein tumor thrombosis; HAICLT, Hepatic arterial infusion chemotherapy combined with lenvatinib plus toripalimab therapy; HAIC, Hepatic arterial infusion chemotherapy. Bold values indicate a statistically significant covariate.

**Figure 3:**
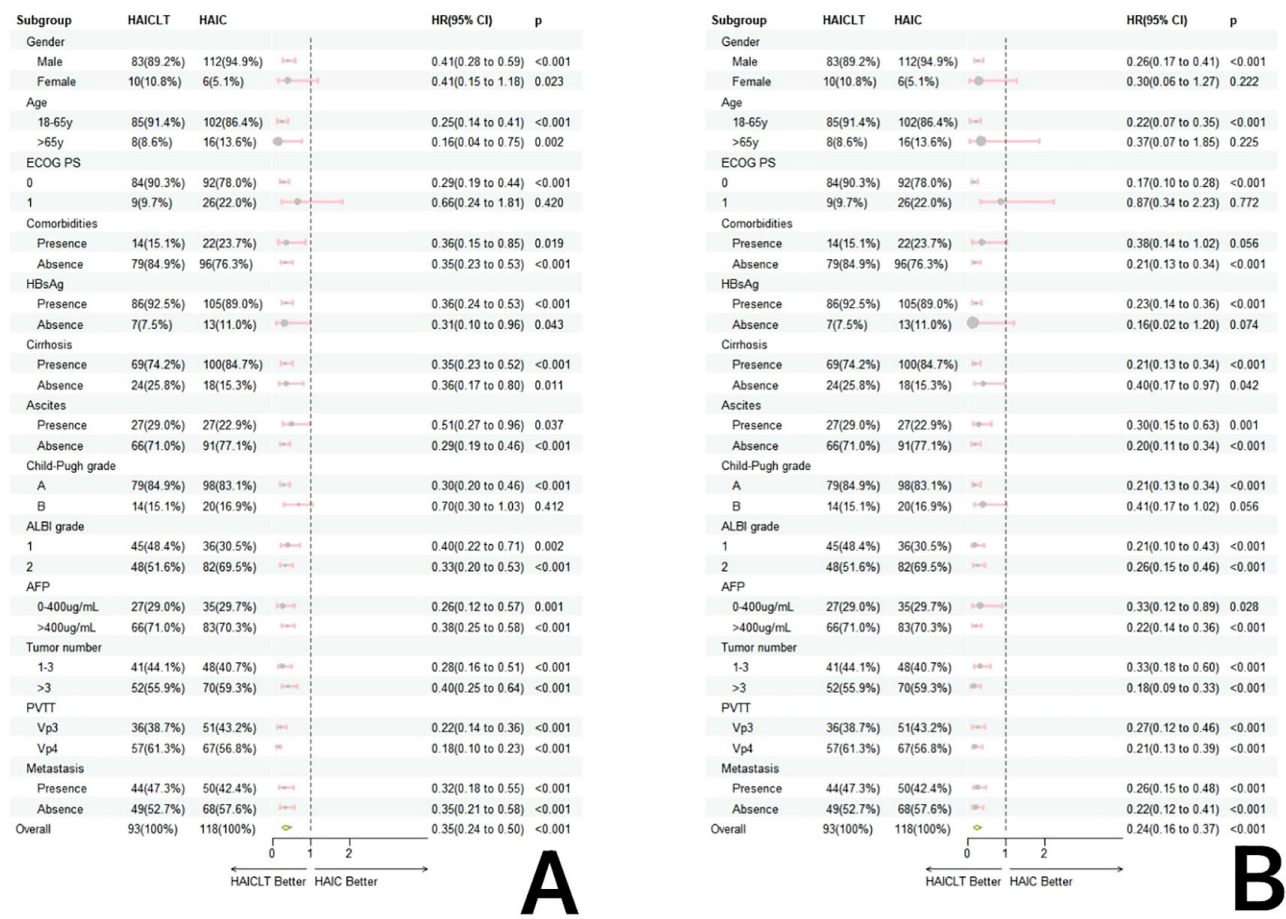
Forestplot based on overall survival **(A)** and progression-free survival **(B)** of each subgroup.

In the subgroup analysis, except for the overall group where the results were affected by the sample size bias, for large HCC with portal vein main trunk tumor thrombus, the combination of HAIC and Lenvatinib plus toripalimab resulted in significantly better survival benefits than HAIC monotherapy. The forest plot of the subgroup analysis is shown in **Figure 3**.

### 3.5 Safety

The incidence rates of any - grade and grade 3–4 adverse reactions are shown in **Table 4**. There were no treatment related deaths during the treatment process. In the HAICLT group, the most common adverse reactions were hypoalbuminemia(59.1%), elevated AST(54.8%), and elevated AST(49.5%). Among them, the most common grade 3–4 adverse reactions were elevated AST (16.2%), abdominal pain (15.1%), elevated AST(12.9%) and hypoalbuminemia(12.9%). In the HAIC group, the most common adverse reactions were hypoalbuminemia(48.3%), elevated AST(45.8%), and elevated AST(40.7%). Among them, the most common grade 3–4 adverse reactions were elevated AST(9.3%), abdominal pain(9.3%), nausea(8.5%). Although the incidences of any-grade and grade 3–4 adverse reactions such as liver function impairment, abdominal pain, and fever in the HAICLT group were higher than those in the HAIC group, there were no significant statistical differences in treatment-related adverse reactions between the two groups.

**Table 4:** Treatment-related adverse events.

Adverse events	Any grade			Grade 3/4		
	HAICLT group (n=93)	HAIC group (n=118)	P value	HAICLT group (n=93)	HAIC group (n=118)	P value
AEs-related treatment interruption or dose reduction						
HAIC discontinuation	0(0%)	0(0%)	1.000	0(0%)	0(0%)	1.000
Toripalimab discontinuation	9(9.7%)	NA	–	7(7.5%)	NA	–
Lenvatinib discontinuation	14(15.1%)	NA	–	11(11.8%)	NA	–
Toripalimab-lenvatinib discontinuation	3(3.2%)	NA	–	3(3.2%)	NA	–
HAIC interruption or dose reduction	28(30.1%)	24(20.3%)	0.102	19(20.4%)	15(12.7%)	0.130
Lenvatinib interruption or dose reduction	48(51.6%)	NA	–	32(34.4%)	NA	–
Treatment-related AEs						
Hypertension	30(32.3%)	28(23.7%)	0.168	9(9.7%)	6(5.1%)	0.197
Diarrhea	21(22.6%)	18(15.3%)	0.173	5(5.4%)	3(2.5%)	0.285
Nausea	28(30.1%)	23(19.5%)	0.074	8(8.6%)	10(8.5%)	0.974
Vomiting	22(23.7%)	18(15.3%)	0.122	4(4.3%)	3(2.5%)	0.479
Weight loss	12(12.9%)	10(8.5%)	0.296	3(3.2%)	1(0.8%)	0.212
Fatigue	5(5.4%)	4(3.4%)	0.478	0(0%)	0(0%)	1.000
Fever	24(25.8%)	23(19.5%)	0.274	6(6.5%)	8(6.8%)	0.670
Abdominal pain	39(41.9%)	41(34.7%)	0.285	14(15.1%)	11(9.3%)	0.201
Neurologic toxicity	19(20.4%)	15(12.7%)	0.130	5(5.4%)	2(1.7%)	0.138
Rash	14(15.1%)	9(7.6%)	0.068	0(0%)	2(1.7%)	0.207
Hand-foot syndrome	12(12.9%)	11(9.3%)	0.407	6(6.5%)	4(4.2%)	0.299
Elevated ALT	46(49.5%)	48(40.7%)	0.202	12(12.9%)	7(5.9%)	0.079
Elevated AST	51(54.8%)	54(45.8%)	0.191	15(16.2%)	11(9.3%)	0.053
Anemia	30(32.3%)	32(27.1%)	0.416	9(9.7%)	4(4.2%)	0.135
Leukopenia	24(25.8%)	19(16.1%)	0.082	6(6.5%)	3(2.5%)	0.163
Neutropenia	18(19.4%)	14(11.9%)	0.132	5(5.4%)	3(2.5%)	0.285
Thrombocytopenia	26(28.0%)	24(20.8%)	0.196	8(8.6%)	8(6.8%)	0.620
Hypoalbuminemia	55(59.1%)	57(48.3%)	0.117	12(12.9%)	8(6.8%)	0.132
Hyperbilirubinemia	36(38.7%)	36(30.5%)	0.212	10(10.8%)	7(5.9%)	0.202
Elevated creatinine	13(14.0%)	10(8.5%)	0.203	2(2.2%)	2(1.7%)	0.810
Hypothyroidism	7(7.5%)	6(5.1%)	0.464	1(1.1%)	0(0%)	0.259
Proteinuria	8(8.6%)	8(6.8%)	0.620	0(0%)	0(0%)	1.000

HAICLT, Hepatic arterial infusion chemotherapy combined with lenvatinib plus toripalimab therapy; HAIC, Hepatic arterial infusion chemotherap; ALT, Alanine aminotransferase; AST, Aspartate aminotransferase.

## 4 Discussion

This multicenter, retrospective study indicates that the combination of HAIC, lenvatinib, and toripalimab has achieved remarkable efficacy in the treatment of large HCC with major PVTT. The combination of HAIC with lenvatinib and toripalimab has significantly prolonged the OS by 186% and the PFS by 120% respectively. Moreover, this combination therapy has effectively promoted intratumoral remission(57.7%) and tumor thrombus remission(62.0%). Notably, 15.1% of the patients achieved conversion and underwent surgical resection. These results remained consistent in both the propensity score matching analysis and subgroup analysis.

Previous randomized trials have reported the efficacy of combining HAIC with sorafenib in the treatment of HCC complicated by major PVTT. The median OS reached an encouraging 16.3 months, and half of the patients achieved an objective response, significantly outperforming the 6.5 months median OS of standard sorafenib monotherapy (17). A preliminary exploration by Xu YJ et al. investigated the efficacy of HAIC combined with toripalimab (18). For advanced HCC, HAIC combined with toripalimab significantly improved the survival prognosis compared with lenvatinib. The median OS and median PFS reached 17.1 months and 9.3 months, respectively. Another phase II trial reported the efficacy of toripalimab combined with bevacizumab in the treatment of advanced HCC (19). The median PFS reached 9.7 months and the median OS was not reached, which was superior to the results reported in the previous IMbrave150 and CARES-310 trials (20, 21). Currently, the efficacy of the combination of HAIC, lenvatinib, and toripalimab has been reported in two studies, both yielding positive results. One phase II trial reported an encouraging median PFS of 10.4 months in high-risk HCC patients treated with the combination of HAIC, lenvatinib, and toripalimab (15). The ORR reached 66.7%, which was superior to the results of our present study. This may because the inclusion criteria for high-risk HCC in that trial were rather broad and thus could not accurately reflect the benefits for patients with both high tumor burden and PVTT. Another retrospective study explored the application of the combination of HAIC, lenvatinib, and toripalimab in advanced HCC. Although the median OS was not reached, the median PFS was an encouraging 11.1 months, and the ORR reached 67.6% (16).

Of particular note, in the subsequent study of the IMbrave150 trial reported at the 2022 American Society of Clinical Oncology(ASCO), the median OS of HCC with PVTT treated with Atezolizumab plus Bevacizumab was only 7.6 months (22), which was far lower than that in this study group. Subsequently, the median OS in the CARES - 310 trial and the HEPATORCH trial reached an encouraging 22.1 months and 20.0 months respectively (19, 21), which was similar to that in this study group. Moreover, in the recent TRIPLET trial, the addition of HAIC to the CARES-310 trial regimen nearly doubled the median progression-free survival and achieved a breakthrough objective response rate of 88.6%, which was significantly higher than the 33.1% reported in the CARES-310 trial, although the median overall survival was not reached (23). These results are superior to those of this study, mainly because the patients included in this study had a higher tumor burden and major PVTT with a poorer prognosis. In addition, given the worse baseline conditions, more consideration needs to be given to the tolerance of liver function, which directly affects the treatment prognosis. On the other hand, this study showed that the surgical conversion rate of HCC larger than 10 cm accompanied by major PVTT treated with HAIC in combination with lenvatinib and toripalimab reached a satisfactory 15.1%. This implies that this triple - therapy regimen has the potential to provide “bridge-to-surgery” for this special population. Meanwhile, these survival benefits need to be verified by future prospective randomized controlled trials.

The superior survival prognosis of the combination therapy may be attributed to the synergistic effects among the three treatments. Firstly, in anti-angiogenesis, HAIC directly damages tumor blood vessels by injecting chemotherapeutic drugs through the intrahepatic target artery, while lenvatinib blocks the angiogenesis signaling pathway by inhibiting multiple targets such as vascular endothelial growth factor receptors (VEGFR) (24). Their combination enhances the anti-angiogenic effect at different stages. Meanwhile, lenvatinib improves the tumor microenvironment, facilitating the killing effect of T-cells activated by toripalimab, and enhances tumor cells’ sensitivity to chemotherapy drugs while reducing drug resistance (25). Secondly, in terms of immune activation, HAIC induces immunogenic death of tumor cells to release antigens. Lenvatinib improves the tumor microenvironment, which is beneficial for immune cell infiltration and function. Toripalimab relieves immune suppression. Through their synergy, a complete immune response chain from antigen release, presentation to immune cell activation and killing is formed, comprehensively enhancing the body’s anti-tumor immune capacity (26). Moreover, in terms of inhibiting tumor cell proliferation and metastasis, lenvatinib disrupts tumor cell growth signaling pathways. HAIC, through its local chemotherapeutic effects, damages tumor cell DNA and interferes with cellular metabolism. Combined with the cytotoxic activity of toripalimab-activated immune cells, these agents synergistically suppress tumor cell proliferation (27, 28). Furthermore, the anti-angiogenic effects of Lenvatinib reduce the likelihood of tumor cells entering the bloodstream. Toripalimab activated immune cells eliminate circulating tumor cells. Meanwhile, the control of localized tumors by HAIC reduces the risk of tumor cell shedding and metastasis (29, 30). These mechanisms collectively inhibit tumor progression and metastasis.

Multivariate analysis based on Cox proportional hazards regression revealed that Vp4 type PVTT and receiving only HAIC were risk factors associated with a poorer OS. Meanwhile, Vp4 type PVTT, extrahepatic metastasis, and receiving only HAIC were risk factors associated with a poor PFS. In the subgroup analysis, there was no significant prognostic relevance only in the subgroups of females, patients aged over 65 years, and those with an ECOG performance status of 1. This was mainly due to the relatively small number of cases in these subgroups resulting from population bias.In terms of safety, although the incidence of adverse reactions such as decreased liver function, abdominal pain, and fever was higher in the combination therapy group than in the monotherapy group, there were no statistically significant differences in the incidence of adverse reactions of any grade or of grade 3–4 between the groups. Moreover, these adverse reactions could potentially be regarded as manifestations of the treatment efficacy. Furthermore, the significantly higher incidence of hypertension (32.3% vs. 23.7%), diarrhea (22.6% vs. 15.3%), and hand-foot syndrome (12.9% vs. 9.3%) in the combination therapy group aligns with the characteristic class effects of the VEGFR-targeted inhibitor lenvatinib. The observed trends of rash(15.1% vs. 7.6%) and hypothyroidism(7.5% vs. 5.1%) are consistent with the immune-related toxicity profile of the PD-1 inhibitor toripalimab. In contrast, hematologic toxicities(leukopenia 25.8% vs. 16.1%) and liver function abnormalities(elevated AST 54.8% vs. 45.8%) primarily reflect the cytotoxic effects of HAIC. Notably, the markedly increased incidence of gastrointestinal adverse events such as nausea (30.1% vs. 19.5%) and vomiting (23.7% vs. 15.3%) in the combination group suggests potential synergistic toxic effects resulting from multi-drug therapy.

This study focused on a fragile HCC population with large tumors and major PVTT, in whom hepatic decompensation and immune-related adverse events(irAEs) are virtually unavoidable and directly impact treatment tolerance. In the HAICLT combination therapy group, 9 patients(9.7%) discontinued toripalimab due to irAEs. However, indicators of hepatic dysfunction were both more prevalent and severe: any-grade hypoalbuminemia occurred in 59.1% of patients, hyperbilirubinemia in 38.7%, while Grade 3/4 transaminase elevations(ALT 12.9%, AST 16.2%) and hyperbilirubinemia(10.8%) also represented substantial proportions. These manifestations led to significantly more frequent interruptions or dose reductions of HAIC(30.1%) and lenvatinib(51.6%). These data demonstrate that although irAEs leading to toripalimab discontinuation were not uncommon in the combination therapy group, hepatic dysfunction reflected by abnormal liver laboratory parameters was both more pervasive and severe among patients.

The present research is not without its constraints. To begin with, although PSM was employed to mitigate the baseline disparities among groups, the innate differences arising from the retrospective nature of the study are still inevitable. Given the retrospective design, it is challenging to entirely eliminate the pre-existing variations between the groups, which could potentially influence the research outcomes. Secondly, due to the distinctive features of the study population, the number of enrolled patients is relatively small. This limited sample size might result in a skewed data distribution during specific subgroup analyses. With a small number of participants, the representativeness of the data within certain subgroups may be compromised, leading to less reliable statistical inferences. Moreover, this study mainly focuses on hepatitis B virus(HBV) related HCC. As alcohol related HCC is more prevalent in Western regions, the generalizability of the findings to these areas requires further investigation. The differences in the etiology of HCC between different regions imply that the results obtained from a study on HBV related HCC may not be directly applicable to Western populations where alcohol - related HCC is the dominant form. In the future, large-scale, international randomized controlled trials are still essential to further corroborate these findings. Furthermore, this study did not adjust for several potential confounders, such as comorbidities, liver functional reserve, differences in supportive care and other molecular or genomic features. Future clinical studies with more comprehensive baseline characterization are required to validate our findings.

In summary, for large HCC with major PVTT, HAIC combined with lenvatinib plus toripalimab can effectively relieve intrahepatic lesions and PVTT with tolerable safety and is a promising treatment option.

## Data Availability

The raw data supporting the conclusions of this article will be made available by the authors, without undue reservation.
